# A study to estimate tooth size discrepancy values specific to Saudi orthodontic patients: A systematic review and *meta*-analysis

**DOI:** 10.1016/j.sdentj.2023.03.006

**Published:** 2023-03-21

**Authors:** Hamad Alqahtani

**Affiliations:** Orthodontic department, faculty of dentistry, King Abdulaziz university, Jeddah 21589, Saudi Arabia

**Keywords:** Odontometry, Malocclusion, Orthodontics, Meta-analysis, Diagnosis, Systematic review

## Abstract

**Objective:**

This study aimed to establish intermaxillary tooth size ratios (overall index (OI) and anterior index (AI)) for normal occlusion and different Angle’s malocclusions specific to the Saudi population and compare them to Bolton’s values, which were specific to Americans.

**Methods:**

The Saudi Digital Library, PubMed, Web of Science, Cochrane Library, Scopus, and Embase were searched systematically to acquire articles which reported OI and AI for Saudi patients; inclusion and exclusion criteria were applied. Data, including author’s name, publication year, city, measurement technique, number of subjects, type of occlusion, means, and standard deviations, were extracted and sorted. To assess the methodological quality of the included studies, The National Heart, Lung, and Blood Institute Quality Assessment Tool for Observational Cohort and Cross-Sectional Studies was utilized.

**Results:**

Of the 1473 articles, eight met the inclusion criteria and were included in the *meta*-analysis. Values for normal occlusion and Bolton’s values were not significantly different. All malocclusion classes showed significant differences in the overall OI and AI values compared with Bolton’s original values and values for normal occlusion, but they did not differ from each other. Gender showed an effect only on the OI ratio for Class III malocclusion.

**Conclusions:**

Bolton’s original values can be used in Saudi patients only with normal occlusion. For cases with any Angle’s malocclusion, we recommend using an AI value of 79.08 (±3.4) for both genders. Classes I and II, regardless of gender, have the same OI value of 92.51 (±2.82). For Class III, values of 91.97 (±2.4) for females and 93.13 (±2.6) for males can be used.

## 1 Introduction

To achieve a proper interdigitation, overjet, overbite, function, and aesthetics, maxillary and mandibular teeth should have proportionally balanced mesiodistal widths (Bolton 1958). The intermaxillary tooth width ratio was first studied in 1923 by (Young 1923), followed by (Gilpatric 1923), who found that maxillary teeth were 8–12 mm wider than the mandibular teeth. Since then, although many methods have been developed to evaluate the relationship between the total mesiodistal width of the maxillary and mandibular teeth, Bolton’s method has become popular in the orthodontic literature (Machado et al., 2020). To estimate the ratio between the sums of the mesiodistal widths of the maxillary and mandibular teeth, Bolton created an overall index (OI) and anterior index (AI). OI was defined as the percentage of the total mesiodistal widths of 12 mandibular teeth (first molar to first molar) to 12 maxillary teeth, which was reported to be 91.3% (±1.91). AI is defined as the percentage of the total mesiodistal widths of six lower anterior teeth to six maxillary anterior teeth, which was reported to be 77.2% (±1.65). Therefore, a resultant percentage greater than these values indicates an increased mandibular tooth size (Bolton, 1958, Bolton, 1962).

Bolton’s ratios have some limitations. They were established based on a group of 55 subjects with excellent occlusion. Forty-four patients were treated orthodontically, and 11 were untreated. Bolton did not specify the gender or ethnic background of the included cases (Bolton 1958). Therefore, these values cannot be applied as standard measures in all populations. Moreover, because his ratios were based on ideal occlusion cases, these values are unrealistic for applications to different malocclusion cases. Thus, many studies have investigated Bolton’s ratios and reported different values among different ethnic groups, populations, genders, and malocclusions (Alshahrani et al., 2020, Machado et al., 2020, Mollabashi et al., 2019). Several studies have been conducted in the Saudi population to measure tooth size discrepancy and investigate the prevalence of Bolton discrepancy (Al Sulaimani and Afify, 2006, Aldrees et al., 2015, Alshahrani et al., 2020, Asiry and Hashim, 2012, Omar et al., 2018). In all these studies, Bolton’s values, which were based on American patients, were referred to when Saudi subjects were included.

A *meta*-analysis is described as a statistical analysis that integrates findings from results obtained by multiple independent studies focused on the same question. It aims to create a weighted average using quantitative methods by consolidating statistical measures from two or more studies. This facilitates data pooling to reach a general conclusion (Aldrees 2011).

The aim of this study was to perform a systematic review and collect data from all studies that investigated tooth size discrepancy among Saudi subjects. Furthermore, it aimed to reach a consensus between articles that have previously reported different Bolton’s values by calculating specific values for Saudis reported in these articles. As these articles considered a relatively small number of subjects, a *meta*-analysis was conducted to acquire a larger sample size and obtain significant statistical support for results pertaining to specific ratios for Saudi patients.

## 2 Materials and methods:

### 2.1 Protocol and search strategy

The protocol for this study was registered with PROSPERO (ID number: CRD42022298665). To collect studies related to Bolton’s analysis and tooth size discrepancy among Saudi patients, a systematic search was undertaken using the following databases: Saudi Digital Library, PubMed, Web of Science, Cochrane Library, Scopus, and Embase. References cited in the acquired articles were manually searched to include all relevant studies. The following subject headings were used: [‘Saudi tooth-size ratios’ OR ‘Saudi tooth-size discrepancy’ OR ‘Saudi Bolton ratio’ OR ‘Saudi Bolton analysis’ OR ‘Saudi tooth-size measurement’ OR ‘Saudi Bolton discrepancy’]. Furthermore, unpublished Master’s theses submitted to the Orthodontic Department, King Abdulaziz university, were searched using the same subject headings.

### 2.2 Eligibility

The population, exposure, comparison, and outcome (PECO) criteria were utilized to assess the suitability of the selected articles, based on the inclusion and exclusion criteria. The study population was defined as Saudi orthodontic patients whose dental casts were obtained at dental clinics or schools. Exposure was measurement and calculation of intermaxillary tooth size discrepancy. The comparison was to Bolton’s original values, gender, and malocclusions. The outcomes were the intermaxillary overall and anterior tooth size ratios specific to Saudi patients.

The eligibility of each article to be included in this study was based on the following criteria: English-language studies; studies including only Saudi orthodontic patients; clear description of sample, Angle’s malocclusion, and measurements; provision of numerical values for intermaxillary tooth size discrepancy (mean and standard deviation (SD)); cross-sectional studies; human study population; the use of physical or digital casts; presence of permanent maxillary and mandibular incisors, canines, premolars, and first molars; no tooth deformities that might affect mesiodistal dimension, including restorations, caries, or abrasion; and the use of a software or digital caliper to measure the teeth mesiodistally to the nearest 0.01 mm.

The exclusion criteria included case reports, narrative reviews, case series studies, studies on cases with missing teeth, and studies missing one of the numerical values (mean or SD).

### 2.3 Quality assessment of included studies

To assess the methodological quality of the included articles, we utilized “The Quality Assessment Tool for Observational Cohort and Cross-Sectional Studies statement proposed by the National Heart, Lung, and Blood Institute (NIHLBI)” (Higgins et al., 2003). The checklist of this tool contains 14 criteria that require verification. The modification of this tool by (Machado et al., 2020) was followed in this analysis because criteria 7, 8, 10, and 13 were not applicable. The checklist was modified to include 10 points in total, with each criterion being assigned one point. Articles scoring 10 or 9, 8 or 7, and 6 or less were considered of high, medium, and low quality respectively. Following the Cochrane Handbook, low-quality studies were excluded from the analysis.

### 2.4 Data extraction and management

Data were retrieved and sorted in a table under the following headlines: author’s name, publication year, city, measurement technique, number of subjects, type of occlusion (normal occlusion, Angle’s Class I, Class II, or Class III), number of females and males, mean OI with SD, mean AI with SD, OI mean values with SD for females and males, AI mean values with SD for females and males, and findings.

### 2.5 Strategy for data synthesis

To achieve the aim of the study, two strategies were followed: the first one was pooling values of OI and AI for different occlusions regardless of gender: comparison of each occlusion value with Bolton’s original values, comparison of different malocclusions with normal occlusion values, and comparison of values among malocclusions. The second strategy was pooling gender-specific OI and AI values in each class of occlusion: comparison of pooled gender-based OI and AI in each class of occlusion and of occlusion groups within the same gender.

To accomplish the first strategy, values of OI and AI regardless of gender were pooled for each occlusion’s category. For normal occlusion, the data were pooled from (Taibah 2016), (Alkofide and Hashim 2002), (Al-Tamimi and Hashim 2005), and (Alshahrani et al., 2020). For Class I and III, (Al Sulaimani and Afify, 2006) and (Alkofide and Hashim 2002) were included. For Class II, (Al Sulaimani and Afify, 2006), (Alkofide and Hashim 2002), and (Asiry and Hashim 2012) were included.

For the second strategy, OI and AI values were pooled for each occlusion’s category and assigned to the male and female categories. For normal occlusion, the data were pooled from (Al-Tamimi and Hashim 2005), (Alshahrani et al., 2020), and (Murshid 2013). For Class I and III, the data from (Alkofide and Hashim 2002), (Omar et al., 2018), and (Alshahrani et al., 2020) were pooled. For Class II, the data from (Alkofide and Hashim 2002), (Omar et al., 2018), (Alshahrani et al., 2020), and (Asiry and Hashim 2012) were included.

## 3 Statistical methods

“Two-independent samples T-test” and “one-Way ANOVA” were used to calculate statistical differences between the results of the included studies. All *p* values less than 0.05 were considered statistically significant.

## 4 Results

**Fig. 1:**
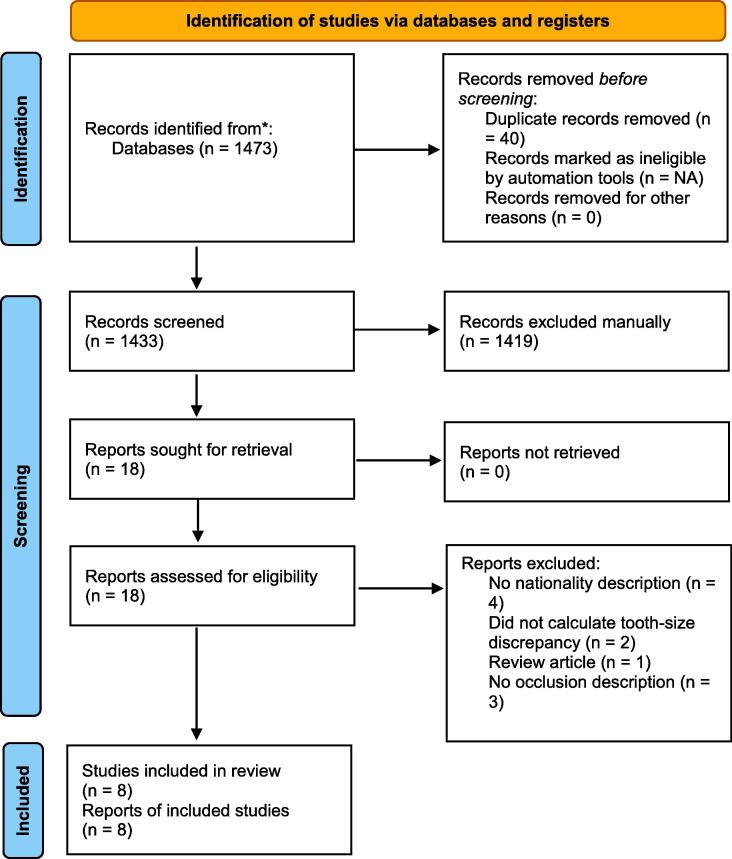
PRISMA flow diagram.

We retrieved 1473 articles through electronic literature search. Forty duplicates and 1419 non-relevant articles were excluded. Eighteen full-text articles remained and were assessed for their eligibility. Ten articles were further excluded based on exclusion criteria. Therefore, eight full text articles were included in the analysis (Fig. 1). A detailed description of the included studies with their reported means, SD, and major findings is given in (Table 1). Based on the Quality Assessment Tool proposed by the NIHLBI, all studies were categorized as medium quality. Only one article scored 8, whereas the rest scored 7.

**Table 1:** Studies included in the *meta*-analysis.

Authors	Year	City	Technique	Subjects (N)	Occlusion	Female /Male	Total OI (SD)	TotalAI (SD)	Female / MaleOI (SD)	Female / MaleAI (SD)	Findings
Alshahrani (Alshahrani et al., 2020)	2020	Abha	Meshmixer™ software(Digitally scanned casts)	120	Total	60/60	NS	NS	NS	NS	* No sig diff between genders ***or*** malocclusions * Malocclusion ratios are sig higher when compared to Bolton * No significant differencein OR of the control group with but “AR” was sig higher than Bolton’s
				30	* Normal occlusion	15/15	92.02 (2.02) Combined normal	78.23 (2.52) Combined normal	91.78 (1.99)/ 92.08 (2.13)	77.94 (2.29)/ 78.51 (2.79)	
				30	* Class I	15/15	92.01 (1.98) Total for combined malocclusion groups	78.61 (2.96) Total for combined malocclusion groups	91.07 (1.91)/ 92.54 (2.10)	78.34 (3.83)/ 79.08 (2.51)	
				30	* Class II	15/15			91.83 (2.14)/ 92.33 (1.92)	78.18 (2.81)/ 78.70 (2.85)	
				30	* Class III	15/15			92.02 (2.39)/ 92.85 (2.26)	78.61 (3.37)/ 79.20 (3.70)	
Omar (Omar et al., 2018)	2018	Jeddah	Digital caliper(Casts)	149	Total	85/64	92.21 (3.66)	79.81 (5.42)	92.37 (3.90)/ 92.00 (3.30)	79.51 (5.30)/ 80.19 (5.60)	* No sig diff between genders ***or*** malocclusion * Ratios are sig increased when compared to Bolton
				81	* Class I mal		NS	NS	92.28 (4.42)/ 91.16 (3.26)	79.91 (6.08)/ 78.58 (4.32)	
				49	* Class II		NS	NS	92.82 (2.88)/ 92.32 (2.71)	79.14 (3.07)/ 80.68 (3.16)	
				19	* Class III		NS	NS	91.46 (2.73)/ 93.63 (3.46)	78.18 (4.93)/ 83.56 (7.64)	
Taibah (Taibah 2016)	2016	Jeddah	Digital caliper(Casts)	84	Open bite	42/42	90.46 (3.90)	77.14 (3.27)	89.97 (3.88)/ 91.48 (2.91)These values are for combined occlusion groups	76.81 (3.24)/ 77.50 (2.91)These values are for combined occlusion groups	* No sig diff bet control and open bite in regard to total OI & AI * OI had significant difference only between male and female open bite subjects * No sig diff with Bolton study
				33	Control ideal occlusion	18/15	91.15 (1.95)	76.97 (2.26)			
Murshid (Murshid 2013)	2013	Jeddah	Digital caliper(Casts)	70	Normal occlusion	35/35	NS	NS	91.60 (1.92)/ 91.79 (1.81)	77.75 (1.75)/ 77.92 (1.99)	* No sig diff between genders * No sig diff with Bolton study
Asiry (Asiry and Hashim 2012)	2012	Riyadh	Digital caliper(Casts)	60	Class II div 1	30/30	91.93 (2.08)	77.65 (2.79)	91.79 (2.13)/ 92.08 (2.05)	77.41 (3.13)/77.90 (2.13)	* No sig diff between genders * No sig diff with Bolton study * No sig difference when compared to class I normal occ (Al-Tamimi)
Al Sulaimani (Al Sulaimani and AR 2006)	2006	Jeddah	Ortho-l software(Digitally scanned casts)	160	Total	98/62	NS	NS	94.10 (3.34)/ 93.27 (4.60)These values are for combined occlusion groups	82.02 (4.78)/80.24 (4.50)These values are for combined occlusion groups	* No statistically significant differences in OI & AI among different occl ***or*** gender * No comparison with Bolton study
				98	* Class I mal	62/36	93.90 (4.07)	81.11 (5.07)			
				52	* Class II	34/18	93.06 (3.65)	81.88 (4.31)			
				10	* Class III	2/8	96.30 (1.45)	80.58 (3.74)			
Al-Tamimi (Al-Tamimi and Hashim 2005)	2005	Riyadh	Digital caliper(casts)	65	Normal occlusion	28/37	91.04 (1.50)	77.40 (1.85)	91.15 (1.53)/ 91.50 (1.50)	77.30 (1.70)/ 77.60 (1.90)	* No sig diff between genders * No sig diff with Bolton study
Alkofide (Alkofide and Hashim 2002)	2002	Riyadh	Digital caliper(casts)	240	Total	120/120	92.61 (2.04)	78.86 (2.55)	NS	NS	* No sig diff between gender for OI * Sig diff between gender in AI (F with Class III) * Sig diff with Bolton study in OI & AI
				60	* Normal occlusion	(30/30)	93.58 (2.12)	78.86 (2.55)	NS	NS	
				60	* Class I mal	(30/30)	92.24 (2.04)	78.77 (2.74)	92.40 (2.30)/ 92.10 (1.60)	78.80 (3.20)/ 78.80 (2.30)	
				60	* Class II	(30/30)	92.80 (2.20)	78.70 (2.45)	93.10 (2.20)/ 92.50 (2.20)	78.80 (2.20)/ 78.60 (2.70)	
				60	* Class III	(30/30)	92.71 (2.12)	78.50 (2.53)	92.20 (2.00)/ 93.20 (2.20)	77.30 (2.00)/79.70 (2.50)	

N, number.

OI, overall index.

AI, anterior index.

SD, standard deviation.

Of the total sample size of 588 patients, 188 had normal occlusion, 158 had Class I malocclusion, 172 had Class II malocclusion, and 70 had Class III malocclusion. Regarding the first strategy of this analysis, no significant difference was found between values for Saudi patients with normal occlusion and Bolton’s original values. All malocclusion classes showed a significant difference in the overall AI and OI values when compared to Bolton’s original values and values for normal occlusion. Class I, II, and III malocclusions did not show significant differences when compared to each other (Table 2).

**Table 2:** Comparison of Saudi’s different occlusions.

	AI				OI			
	n	Mean	SD	*p* value*	n	Mean	SD	*p* value*
**Comparison of different occlusions with Bolton’s original values**								
Bolton’s Original Value	55	77.20	1.65	-	55	91.3	1.91	-
Normal Occlusion	188	77.74	2.29	0.06	188	91.75	1.89	0.132
Class I	158	79.30	3.9	<0.001*	158	92.57	3.0	<0.001*
Class II	172	78.80	3.18	<0.001*	172	92.45	2.64	<0.001*
Class III	70	79.15	3.13	<0.001*	70	94.84	1.78	<0.001*
**Comparison of class (I, II and III) with the normal occlusion**								
Normal occlusion	188	77.74	2.29	-	188	91.75	1.89	-
Class I	158	79.30	3.9	<0.001*	158	92.57	3.0	0.003*
Class II	172	78.80	3.18	<0.001*	172	92.45	2.64	0.004*
Class III	70	79.15	3.13	<0.001*	70	94.84	1.78	<0.001*
**Comparison of different malocclusions**								
Class I	158	79.30	3.9		158	92.57	3.0	
Class II	172	78.80	3.18		172	92.45	2.64	
Class III	70	79.15	3.13		70	94.84	1.78	
*p* value**	0.95				0.42			

AI, Anterior Index; OI, Overall Index; SD, standard deviation; n, sample size.

*p*: significance level (*p* < 0.05).

* Two Independent samples T-test.

** One-way ANOVA.

With regard to the second strategy of our analysis, no significant difference was found between and within genders in each class and between classes of malocclusion for the overall OI and AI values, except for Class III malocclusion, in which males had higher values than females (*p* = 0.0068), and all males in other occlusion classes (*p* = 0.009), as shown in (Table 3).

**Table 3:** Comparison of Saudi’s gender-specific overall OI and AI values in different occlusion classes.

AI								OI						
Occlusion	Female			Male				Female			Male			
	n	Mean	SD	n	Mean	SD	*p* value	n	Mean	SD	n	Mean	SD	*p* value
Normal	78	77.61	1.9	87	77.90	2.2	0.174	78	91.45	1.8	87	91.72	1.8	0.16
Class I	45	78.79	4.37	45	78.88	3.04	0.353	45	91.68	2.9	45	92.12	2.32	0.489
Class II	75	78.46	2.8	75	78.71	2.7	0.219	75	92.32	2.3	75	92.30	2.2	0.415
Class III	45	77.70	3.4	45	79.82	4.6	0.061	45	91.97	2.4	45	93.13	2.6	0.0068*
*p* value		0.161			0.140				0.313			0.009*		

AI, Anterior Index; OI, Overall Index; SD, standard deviation; n, sample size.

*p*: significance level (*p* < 0.05).

Two Independent samples T-test was used for the row wise comparison.

One-way ANOVA was used for the column wise comparison.

**Fig. 2:**
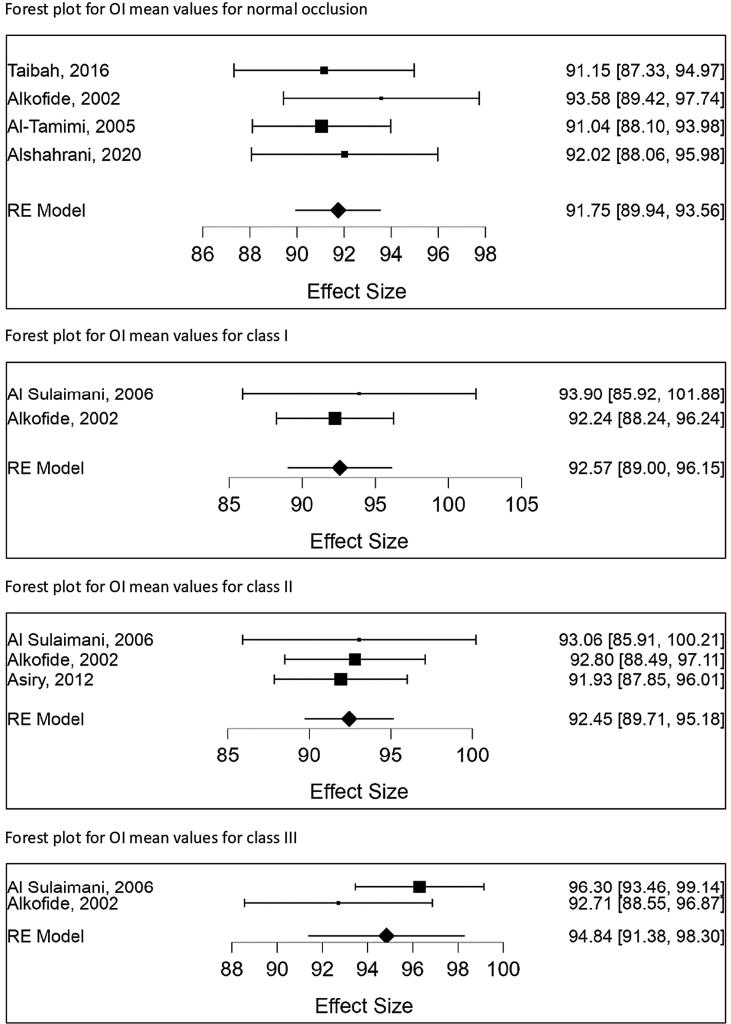
Forest plot for studies with OI means values for Saudi patients. Mean effect size has been calculated with 95% confidence interval CI.

**Fig. 3:**
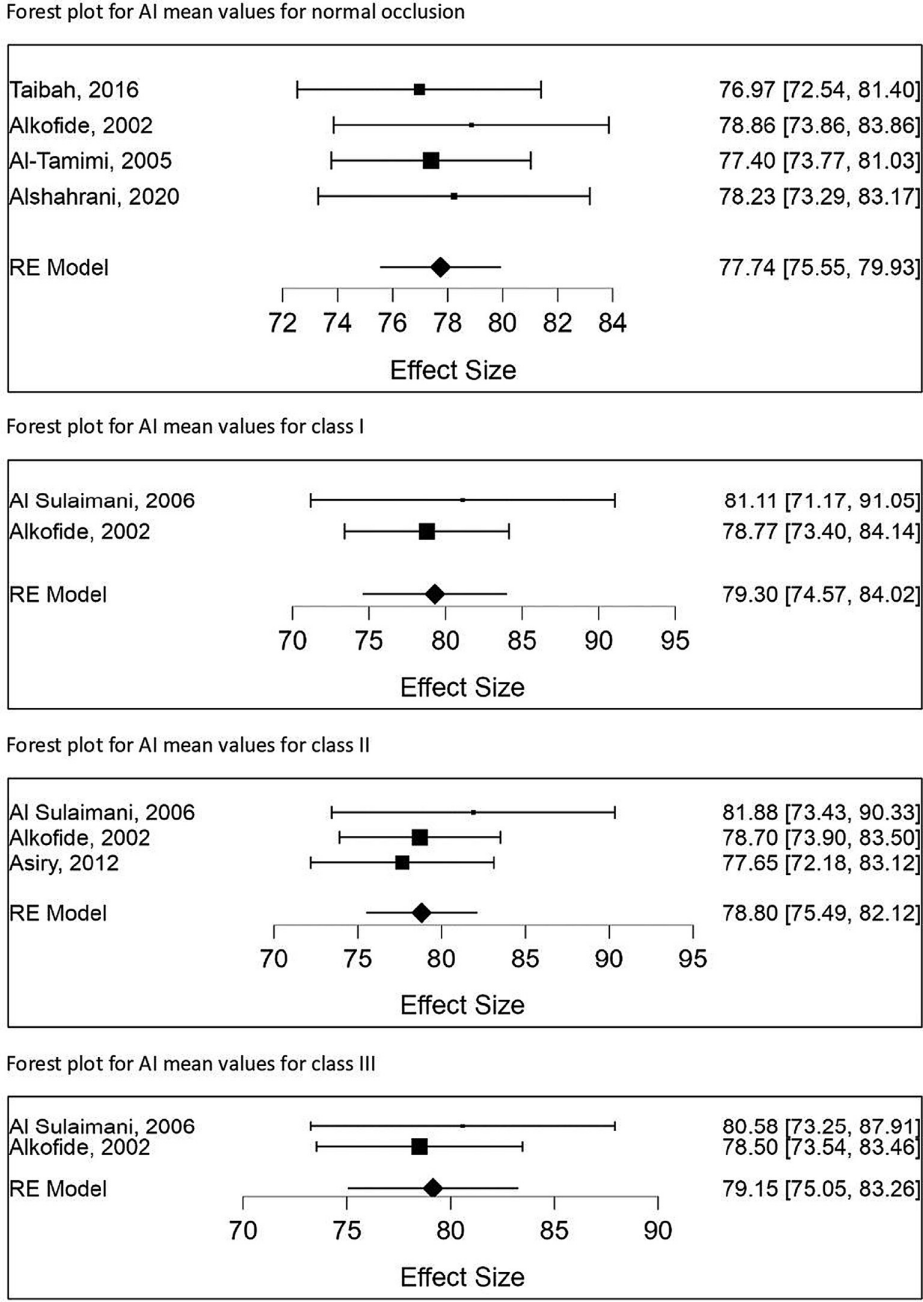
Forest plot for studies with AI means values for Saudi patients. Mean effect size has been calculated with 95% confidence interval CI.

Forest plot for studies with OI and AI means values for Saudi patients are shown in (Fig. 2, Fig. 3) respectively.

## 5 Discussion

This is the only study in the literature that has aimed to estimate AI and OI values specific to Saudi orthodontic patients. Since our results did not show a significant difference in AI and OI values between Bolton’s and normal occlusion values, Bolton’s original values can be used for assessment in Saudi orthodontic patients with normal occlusion only. Similar findings have been reported in Portuguese (Machado et al., 2018), Syrian (Nourallah et al., 2005), Yemeni (Al-Gunaid et al., 2012) and Emirati patients (Mohammad et al., 2018). In contrast to our findings, previous studies have reported a significant difference between values for normal occlusion and Bolton’s values in Iran (Mollabashi et al., 2019) and Finland (Turtinen et al., 2021). Moreover, a recent global *meta*-analysis by (Machado et al., 2020) found that normal occlusion has higher AI and OI values than Bolton’s values.

Since AI and OI values for the malocclusion groups were higher than those for normal occlusion and Bolton’s values but did not differ significantly among themselves, we do not recommend using Bolton’s values in cases with malocclusion. In line with our results, some reports have indicated significant differences between values for malocclusion and Bolton’s values in Portuguese (Machado et al., 2018) and Polish (Wędrychowska-Szulc et al., 2009) subjects. Similarly, (Machado et al., 2020) found a larger OI value in Class I and larger OI and AI values in Class III malocclusions than in normal occlusion subjects. In contrast, other studies have found no significant differences among occlusion groups in Brazilian (Ricci et al., 2013), Nepalese (Mishra et al., 2019), Jordanian (Al-Khateeb and Abu Alhaija 2006) and Turkish (Uysal and Sari 2005) subjects.

Gender-based variations in AI and OI values have been reported in the literature in different countries. Some studies have found that males tend to have larger ratios than females (Al-Gunaid et al., 2012, Smith et al., 2000, Uysal and Sari, 2005, Wędrychowska-Szulc et al., 2009). Other studies have reported no significant differences in these values among Egyptian (Hussein et al., 2022), Syrian (Nourallah et al., 2005), Turkish (Basaran et al., 2006), Yemeni (Al-Gunaid et al., 2012), Iranian (Kachoei et al., 2011), Chinese (Nie and Lin 1999), Nigerian (Adeyemi et al., 2010), Japanese (Endo et al., 2007), Portuguese (Machado et al., 2018), Brazilian (Araujo and Souki 2003) and Spanish (Paredes et al., 2006) subjects. Our results indicate that males with Class III malocclusion had higher values than females with the same malocclusion and males with other occlusion classes. On a global scale, (Machado et al., 2020) reported that males with class I malocclusion tended to have higher values.

Based on our findings, and since there were no malocclusion-based and gender-based variations between AI ratios, we combined all malocclusions AI values to find an average AI value that can be used specifically for Saudi patients with any type of malocclusion. Thus, we suggest using an AI value of 79.08 (±3.4) for cases with malocclusion, irrespective of malocclusion type and gender.

With regard to OI values, we combined Class I and II values only, because there was no gender-based variation. Thus, we suggest using a value of 92.51 (±2.82) for Class I or II, regardless of gender type. Because of the gender-based variation in Class III, we recommend using 91.97 (±2.4) for females and 93.13 (±2.6) for males.

All articles presented in this study had medium methodological quality. Sample size calculation was not performed in all the studies, which affected the generalization of their results. Two articles (Alshahrani et al., 2020) and (Omar et al., 2018) have specified their sample’s demographics, location, and time period. The error of the method for intra-examiner reliability was reported by all the studies except (Omar et al., 2018). Tooth size measurements were performed directly on dental casts using digital calipers, except for (Alshahrani et al., 2020) and (Al Sulaimani and Afify, 2006), who scanned the models and performed measurements using a computer software. Both measurement methods have been tested for accuracy by many authors, who reported no significant difference between the two methods (Abuhassan and Asiry, 2021, Amuk et al., 2019, Murugesan and Sivakumar, 2020).

This study had some limitations. All the studies included in this analysis covered the western, central, and southern regions of Saudi Arabia. No studies were performed in the eastern or northern regions of the country, which might limit the generalizability of our results. Furthermore, the Saudi population has diverse ethnic and racial backgrounds. Thus, future studies should focus on the eastern and northern regions of the country to explore whether they exhibit different intermaxillary tooth size ratios. In addition, we advocate overcoming the methodological limitations of previous studies by performing sample-size calculations, adequate sample recruitment, and examiner calibration.

## 6 Conclusion

For Saudi orthodontics patients, Bolton’s original values can be applied to cases with normal occlusion only irrespective of the patient gender. Regarding AI, for cases with any Angle’s malocclusion regardless of gender, we recommend using a value of 79.08 (±3.4). Regarding OI, a value of 92.51 (±2.82) can be used for Class I and II only regardless of gender. For Class III, values of 91.97 (±2.4) can be used for females and 93.13 (±2.6) for males, respectively.

## Declaration of Competing Interest

The author declares that he has no known competing financial interests or personal relationships that could have appeared to influence the work reported in this paper.

## References

[b0005] Abuhassan S.M., Asiry M.A. (2021). Intermaxillary tooth-size ratios in Saudis: a systematic review. J. Int. Oral Health.

[b0010] Adeyemi A.T., Bankole O.O., Denloye O.O. (2010). Tooth size ratios of Nigerian and the applicability of Bolton's analysis. Odontostomatol. Trop..

[b0015] Al Sulaimani F., Afify A.R. (2006). Bolton analysis in different classes of malocclusion in a Saudi Arabian sample. Egypt Dent. J..

[b0020] Aldrees A.M. (2011). Lateral cephalometric norms for Saudi adults: a meta-analysis. Saudi Dent J..

[b0025] Aldrees A.M., Al-Shujaa A.M., Alqahtani M.A. (2015). Is arch form influenced by sagittal molar relationship or Bolton tooth-size discrepancy?. BMC Oral Health.

[b0030] Al-Gunaid T., Yamaki M., Saito I. (2012). Mesiodistal tooth width and tooth size discrepancies of Yemeni Arabians: a pilot study. J. Orthod. Sci..

[b0035] Al-Khateeb S.N., Abu Alhaija E.S.J. (2006). Tooth size discrepancies and arch parameters among different malocclusions in a Jordanian sample. Angle Orthod..

[b0040] Alkofide E., Hashim H. (2002). Intermaxillary tooth size discrepancies among different malocclusion classes: a comparative study. J. Clin. Pediatr. Dent..

[b0045] Alshahrani A.A., Alshahrani I., Addas M.K. (2020). The tooth size discrepancy among orthodontic patients and normal occlusion individuals from Saudi Arabia: a three-dimensional scan analysis of diagnostic casts. Contemp. Clin. Dent..

[b0050] Al-Tamimi T., Hashim H.A. (2005). Bolton tooth-size ratio revisited. World J. Orthod..

[b0055] Amuk N.G., Karsli E., Kurt G. (2019). Comparison of dental measurements between conventional plaster models, digital models obtained by impression scanning and plaster model scanning. Int. Orthod..

[b0060] Araujo E., Souki M. (2003). Bolton anterior tooth size discrepancies among different malocclusion groups. Angle Orthod..

[b0065] Asiry M., Hashim H. (2012). Tooth size ratios in Saudi subjects with Class II, Division 1 malocclusion. J. Int. Oral Health.

[b0070] Basaran G., Selek M., Hamamci O. (2006). Intermaxillary Bolton tooth size discrepancies among different malocclusion groups. Angle Orthod..

[b0075] Bolton W.A. (1958). Disharmony in tooth size and its relation to the analysis and treatment of malocclusion. Angle Orthod..

[b0080] Bolton W.A. (1962). The clinical application of a tooth-size analysis. Am. J. Orthod..

[b0085] Endo T., Shundo I., Abe R. (2007). Applicability of Bolton's tooth size ratios to a Japanese orthodontic population. Odontology.

[b0090] Gilpatric W.H. (1923). Arch predetermination—Is it practical?. J. Am. Dent. Assoc. (1992).

[b0095] Higgins J.P., Thompson S.G., Deeks J.J. (2003). Measuring inconsistency in meta-analyses. BMJ.

[b0100] Hussein F.A., Mohamed R.E., El-Awady A.A. (2022). Digital evaluation of Bolton's tooth size discrepancies among different malocclusions categories of Egyptian adolescent orthodontic population: a retrospective study. Int. Orthod..

[b0105] Kachoei M., Ahangar-Atashi M.H., Pourkhamneh S. (2011). Bolton's intermaxillary tooth size ratios among Iranian schoolchildren. Med. Oral Patol. Oral Cir. Bucal.

[b0110] Machado V., Botelho J., Pereira D. (2018). Bolton ratios in Portuguese subjects among different malocclusion groups. J. Clin. Exp. Dent..

[b0115] Machado V., Botelho J., Mascarenhas P. (2020). A systematic review and meta-analysis on Bolton's ratios: Normal occlusion and malocclusion. J. Orthod..

[b0120] Mishra R.K., Kafle D., Gupta R. (2019). Analysis of interarch tooth Size relationship in nepalese subjects with normal occlusion and malocclusions. Int. J. Dent..

[b0125] Mohammad M.G., Din S.N., Khamis A.H. (2018). Overall and anterior tooth size ratios in a group of Emiratis. Open Dent. J..

[b0130] Mollabashi V., Soltani M.K., Moslemian N. (2019). Comparison of Bolton ratio in normal occlusion and different malocclusion groups in Iranian population. Int. Orthod..

[b0135] Murshid Z. (2013). Bolton tooth size discrepancy in normal occlusion. Egypt Dent J..

[b0140] Murugesan A., Sivakumar A. (2020). Comparison of accuracy of mesiodistal tooth measurements made in conventional study models and digital models obtained from intraoral scan and desktop scan of study models. J. Orthod..

[b0145] Nie Q., Lin J. (1999). Comparison of intermaxillary tooth size discrepancies among different malocclusion groups. Am. J. Orthod. Dentofac. Orthop..

[b0150] Nourallah A.W., Splieth C.H., Schwahn C. (2005). Standardizing interarch tooth-size harmony in a Syrian population. Angle Orthod..

[b0155] Omar H., Alhajrasi M., Felemban N. (2018). Dental arch dimensions, form and tooth size ratio among a Saudi sample. Saudi Med. J..

[b0160] Paredes V., Gandia J.L., Cibrian R. (2006). Do Bolton's ratios apply to a Spanish population?. Am. J. Orthod. Dentofac. Orthop..

[b0165] Ricci I.D., Scanavini M.A., Kaieda A.K. (2013). Bolton ratio in subjects with normal occlusion and malocclusion. Braz J Oral Sci..

[b0170] Smith S.S., Buschang P.H., Watanabe E. (2000). Interarch tooth size relationships of 3 populations: “does Bolton's analysis apply?”. Am. J. Orthod. Dentofac. Orthop..

[b0175] Taibah S. (2016). Bolton discrepancy among patients with anterior open bite malocclusion. J World Fed Orthod..

[b0180] Turtinen H., Sarja M., Hyvärinen J. (2021). Associations between Bolton ratio and overjet deviations in a Finnish adult population. Acta Odontol. Scand..

[b0185] Uysal T., Sari Z. (2005). Intermaxillary tooth size discrepancy and mesiodistal crown dimensions for a Turkish population. Am. J. Orthod. Dentofac. Orthop..

[b0190] Wędrychowska-Szulc B., Janiszewska-Olszowska J., Stepień P. (2009). Overall and anterior Bolton ratio in Class I, II, and III orthodontic patients. Eur. J. Orthod..

[b0195] Young J.L. (1923). Rational treatment of infraclusion. Int. J. Orthod. Oral Surg. Radiogr..

